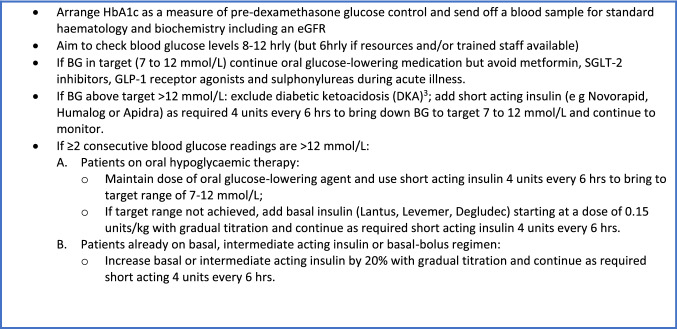# Dexamethasone and oxygen therapy in care home residents with diabetes: a management guide and algorithm for treatment: a rapid response action statement from the European Diabetes Working Party for Older People (EDWPOP) and European Geriatric Medicine Society (EuGMS)

**DOI:** 10.1007/s40520-021-01822-1

**Published:** 2021-04-15

**Authors:** Alan James Sinclair, Stefania Maggi, Ahmed Hassan Abdelhafiz, Nicola Veronese, Leocadio Rodriguez-Manas, Isabelle Bourdel-Marchasson

**Affiliations:** 1 fDROP and King’s College, London, UK; 2 European Diabetes Working Party for Older People (EDWPOP), London, UK; 3 CNR-IN (Aging Branch), Padua, Italy; 4 European Geriatric Medicine Society (EuGMS), Vienna, Austria; 5 Rotherham General Hospital, Rotherham, UK; 6 University of Palermo, Palermo, Italy; 7 University Hospital of Getafe, Madrid, Spain; 8 Chu-Bordeaux, Bordeaux, France

**Keywords:** Long-term care, Diabetes, Oxygen, Dexamethasone, COVID-19

## Abstract

This statement addresses the need to provide clinically relevant and practical guidance for long-term care staff working in care homes and other stakeholders engaged in the care of residents who require consideration for dexamethasone and oxygen therapy. It had been provided following a series of consensus discussions between the EDWPOP and the EuGMS in January and February 2021. Its main aim is to minimise morbidity and mortality from serious acute illnesses including COVID-19 requiring these treatments within the long-term care sector.

This guidance was prepared by two European medical organisations with multidisciplinary input to:

- Alert long-term care facilities (LTC, residential care homes including nursing homes) that they may be expected to manage residents with diabetes with hypoxaemia and pneumonia who require oxygen therapy and/or dexamethasone
- Minimise morbidity and mortality from serious acute illnesses including COVID-19 which require these treatments
- Emphasise that those at the highest risk of poor outcomes from serious acute illness in long-term care are those who have: frailty, several existing medical conditions, such as cardiovascular disease or respiratory disease, diabetes complications, treatment with steroids, a life expectancy < 6 months
- Emphasise that residents requiring oxygen therapy and/or dexamethasone can deteriorate quickly—this is a medical emergency situation

This guidance is addressed to: managers/senior nursing staff of long-term care (LTC) facilities, other care staff, community nursing and diabetes staff, primary care providers including general practitioners.

## Background

The significant impact of COVID-19 on the care home or LTC population has been dramatic in terms of increased morbidity and mortality. In many countries, LTC has been the epicentre of the pandemic, with the incidence and severity of illness and mortality shifted towards older people particularly those with multiple comorbidities, such as diabetes, hypertension, and cardiovascular disease [1]. In addition, a quarter of residents have diabetes and two-thirds may have frailty which is a better predictor of intensive care unit (ICU) outcomes than age or other factors [2].

Residents in LTC are a highly co-morbid population who are particularly vulnerable to developing rapid serious acute illness as has been demonstrated with COVID-19 infection. The huge burden on hospital-based care for managing COVID-19 has meant that many residents with this infection remain in LTC and are not referred to hospital, with care staff being expected to take on additional care not previously considered to be within their remit. This includes administering oxygen and/or dexamethasone therapy.

## Dexamethasone and oxygen therapy

Dexamethasone (a corticosteroid) treatment is associated with improved survival and clinical benefit with people with severe COVID-19 [4]. The results show that dexamethasone reduces 28-day mortality substantially among patients who received oxygen or ventilation at the time of randomisation in the UK Phase 3 open label randomised clinical trial, RECOVERYl [4]. Dexamethasone has a favourable benefit-risk profile when used in those with severe pneumonia. Short-term use (< 2 weeks) is associated with few side effects apart from hyperglycaemia in non-diabetic individuals [5] but may worsen glucose control in those with diabetes mellitus by reducing peripheral use of glucose. It is also important to monitor for fluid and sodium retention.

In LTC, dexamethasone is now also being prescribed when a resident requires oxygen therapy to correct hypoxaemia and maintain satisfactory arterial oxygen saturation (92–96%), unless there is a background of chronic lung disease when a level of > 88% may be more realistic. An oxygenation strategy using high-flow nasal cannulae [6] may be another option that could be considered within a well-resourced LTC setting.

The format for this guide on dexamethasone and oxygen therapy has been developed from earlier UK guidance [3] and designed to support clinical decision-making in long-term care facilities. As such, the guidance will need to be interpreted in the light of the availability of skilled personnel, adherence to local/national regulations for oxygen provision within LTC including monitoring of oxygen delivery and blood oxygen saturation (pulse oximetry), monitoring of blood glucose and ketones, fluid administration limits, and overall level of care that is available. Whenever possible, other usual care practices for a resident with diabetes should be maintained.

## Implementing this guidance

The implementation of this European guidance will require it to be locally adapted and agreed between community-based health professional teams and GP-led services which interact with LTC to provide direct clinical care. Crucially, these treatments require respect of the individual’s values and preferences or that of their family if consent not possible.

Communication between all relevant parties (care homes, community services, primary care, local hospitals) may be enhanced by technology for virtual reviews/case conferences to minimise contacts for healthcare professionals [3]. For residents recently discharged from hospital on dexamethasone, this guidance can still be provide additional advice on continuation of this treatment as recommended by local clinicians/specialists.

## Infection control and prevention

Appropriate infection control precautions should be in place including: Isolation facilities where required, as well as personal protective equipment (PPE). Further information is available at: World Health Organisation (WHO) WHO-2019-nCoV-IPC_long_term_care-2021.1-eng(1).pdf.

This guidance should only be considered when sufficient support is available to safely administer treatments within the LTC/care home environment. Liaison with a respiratory specialist is advisable. This guidance is not to be employed when it is clear that admission to hospital is necessary to avoid further deterioration in the health status of the resident.

## Advice for managers and owners of long-term care facilities


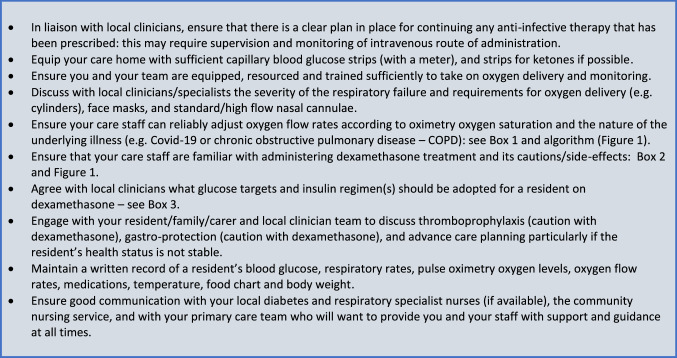


**Fig. 1:**
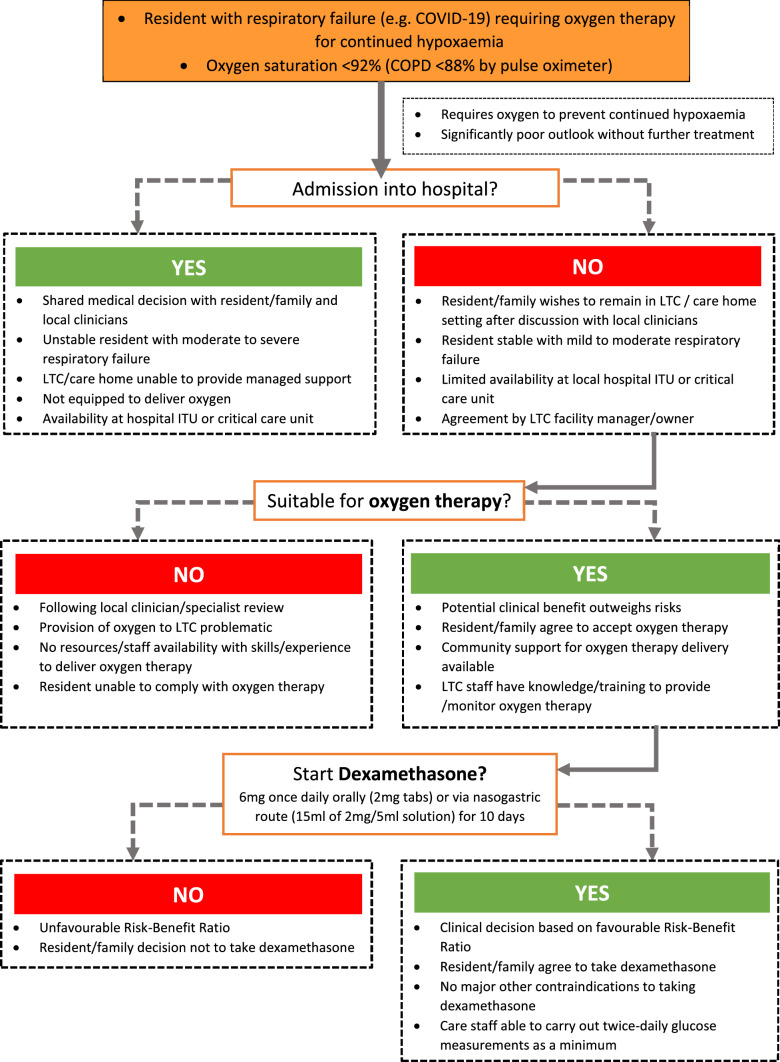
Algorithm for oxygen and dexamethasone therapy in long-term care (LTC)

In Box 1, guidance is given on managing oxygen flow rates via nasal cannulae which will require supervision by a trained nurse or other local clinician initially. In Fig. 1, an algorithm has been provided to manage a resident with mild to moderate hypoxaemia requiring oxygen therapy and dexamethasone using a step-by-step approach. We recognise that some LTC settings will face difficulties in providing this level care.

In Box 2, a set of important clinical alerts has been provided which should be seen as warning alerts that should prompt seeking medical advice and even calling the emergency services in some circumstances.

In Box 3, we provide guidance on the management of glucose levels whilst taking dexamethasone, and suggest insulin regimens that should be considered.

## Box 1: Oxygen flow rate titration guidance


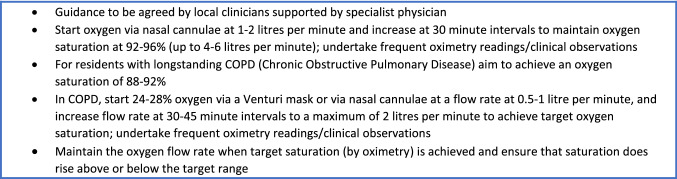


## Box 2: clinical alerts


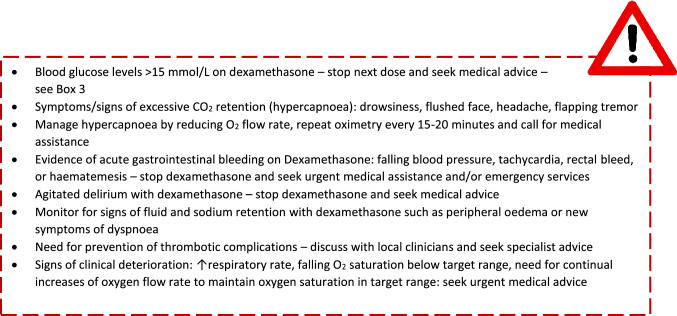


## Box 3: Insulin use in residents with diabetes requiring dexamethasone